# Pilose antler polypeptides promote chondrocyte proliferation via the tyrosine kinase signaling pathway

**DOI:** 10.1186/1745-6673-6-27

**Published:** 2011-11-10

**Authors:** Jian-Hua Lin, Ling-Xiao Deng, Zhao-Yang Wu, Lei Chen, Li Zhang

**Affiliations:** 1 Department of Orthopaedics, First Affiliated Hospital of Fujian Medical University, Fuzhou, Fujian, PR China; 2 Orthopaedics & Traumatology College, Fujian University of Traditional Chinese Medicine, Fuzhou, Fujian, PR China

**Keywords:** PAP, Chondrocyte, Proliferation, S phase, Cyclin A, TK signaling pathway

## Abstract

**Background:**

Pilose antler polypeptides (PAP) have been reported to promote chondrocyte proliferation. However, the underlying mechanism remains unclear. The present study was to investigate the effects of PAP on the proliferation of chondrocytes and its underlying mechanism.

**Methods:**

Chondrocytes isolated from the knee of Zealand white rabbits were cultured. The second generation chondrocytes were collected and identified using safranin-O staining. The chondrocytes were divided into the following 4 groups including serum-free, PAP, genistein (an inhibitor of tyrosine kinases), and PAP plus genistein group. Cell viability was analyzed using the MTT assay. The cell cycle distribution of the chondrocytes was analyzed by flow cytometry. The expression levels of cyclin A was detected using immunocytochemical staining.

**Results:**

No significant difference was observed between serum-free and genistein group. Treatment of the cultures with PAP produced a significant dose-dependent increase in cell viability, the percentage proportion of chondrocytes in the S phase and Cyclin A expression as well. However, the promoting effect of PAP on chondrocyte proliferation were dose-dependently inhibited by genistein, whereas genistein alone had no effect on proliferation of isolated chondrocytes.

**Conclusions:**

The data demonstrate that PAP promotes chondrocyte proliferation with the increased cell number, percentage proportion of chondrocytes in S phase and expression of protein cyclin A via the TK signaling pathway.

## Introduction

The facts that cartilage in deer antler grows at a rate of 1-2 cm per day indicates that some specific regulatory factors in antler tissues may play a key role in promoting the proliferation of chondrocytes. Recently, pilose antler polypeptides (PAP) were developed from velvet antler (VA) of sika deer (Cervus Nippon Temminck), which were found to promote chondrocyte proliferation [1]. However, its underlying mechanism remains obscure.

The proliferation of cells is well regulated by the interactions of a variety of growth factors, cytokines, and signal molecules [2]. Protein kinases, particularly tyrosine kinases (TK) have been characterized as modulating cell proliferation and differentiation [3]. Mitogen-activated protein kinase activation is required for their role as phosphorylating enzymes. These reports led to a hypothesis that TK signaling pathway may be involved in PAP inducing chondrocyte proliferation.

Genistein (4,7,4'-trihydroxyisoflavone), a major isoflavone from soybean, has been proven as a specific inhibitor of TK. Previous studies have confirmed that genistein, which block kinase ATP-binding sites, specifically inhibit phosphorylation of tyrosine residues, thereby inhibiting cells growth [4,5]. As a result, high specificity of genistein has been wide used to study for involvement of tyrosine phosphorylation in cell proliferation.

The present study was to elucidate the effects of PAP on the proliferation of chondrocytes and examine the role of tyrosine phosphorylation in PAP mediating chondrocyte proliferation by using genistein which was expected to inhibit tyrosine phosphorylation in the cell.

## Materials and methods

### Chondrocytes culture and confirmation

The articular cartilages were harvested from the knees joints of one-month-old New Zealand white rabbits (Shanghai animal experimental center), and transferred to phosphate- buffered saline (PBS) with 500 units/ml penicillin and 500 μg/ml streptomycin. Then the cartilages were cut into 1 mm pieces, and digested with 0.25% trypsin/EDTA (Sigma, St. Louis, MO, USA) for 0.5 h and 0.2% collagenase type II (Sigma, St. Louis, MO, USA) subsequently. The isolated cells were collected and counted every 2 hours. They were cultured at a density of 1.5 × 10/ml in F12 culture medium (Sigma, St. Louis, MO, USA) supplemented with 15% fetal calf serum (FCS, Sijiqing Co., Hangzhou, China), and incubated at 37°C in a 5% CO2 incubator. Culture media were changed every 3-4 days, and cells were passaged every week, and the third passages of cells were used in all experiments. After the third passage, cells were harvested and seeded on glass slice for 6 days, and observed under microscopy for the production of glycosaminoglycans (GAG) after Safranin-O staining.

### Experimental design

The cultured chondrocytes were divided randomly into 4 groups: control group, PAP (provided by New Drug Research Center in the Affiliated Hospital of Changchun College of Traditional Chinese Medicine.) group, Genistein (Sigma, St. Louis, MO, USA) group, and PAP + Genistein group. In control group, chondrocytes were cultured with serum-free culture medium only. In PAP group, chondrocytes were cultured with additional PAP at different concentrations, i.e. 0 μg/ml, 12.5 μg/ml, 25.0 μg/ml, 50.0 μg/ml, and 100 μg/ml, respectively. In Genistein group, cells were cultured in medium plus Genistein at 0 μg/ml, 6 μg/ml, 12 μg/ml, 24 μg/ml, and 48 μg/ml. In PAP + Genistein group, cells were cultured with 50.0 μg/ml PAP, as well as Genistein of 0 μg/ml, 6 μg/ml, 12 μg/ml, 24 μg/ml, and 48 μg/ml, respectively.

### MTT assay

Chondrocytes at passage 2 were seeded into 96-well plates at a density of 1 × 104/ml. When cells reached 80% confluences, they were switch to serum-free media for 24 hours. Then 100 μl agent was added into each well with serum-free media, PAP, and Genistein at different concentrations as mentioned above. 48 hours later, 20 μl of 5 g/L (w/v) MTT (Sigma, St. Louis, MO, USA) was added into each well. The cells were incubated for further four hours. Then the supernatant was removed, and 150 μl/well of dimethyl sulphoxide (DMSO) were added to dissolve the formazane. Absorbance was measured at a major wavelength of 570 nm and a reference wavelength of 690 nm with a Reder's plate reader (Reder, Japan) [6]. OD value was obtained for each well.

### Determination of Proliferation by Flow Cytometry

Chondrocytes at passage 2 were seeded into 60 mm culture dishes at a density of 1 × 104/ml and cultured to logarithmic growth phase. Then the cells were switched to serum free F12 culture medium for 24 hours. After that, cells were divided into three groups which serum-free media, 50 μg/ml PAP, and 50 μg/ml PAP plus 48 μg/ml Genistein were added into each group respectively. The PAP should be added 30 minutes after the addition of Genistein which were kept in warm water. Cells were collected 48 hours later, and their densities were adjusted to 105/ml before they were fixed in 75% cold ethanol and incubated at 4°C overnight. The next day, cells were stained by propidium iodide containing 100 mg/ml RNase A before flow cytometric analysis [7].

### Cyclin A Immunocytochemistry

Chondrocytes at passage 2 and 3 at a density of 1 × 106/ml were seeded onto glass slices, which were put inside 6-well plate. 12 hours later when all cells were fully attached on the slices, different cultured media including regular culture media, PAP, PAP with Genisein at different concentrations were added and cultured for 48 hours followed standard immunocytochemical staining procedure. Afterwards, the slides were treated for 15 min in 0.1% Triton X-100 in PBS, rinsed in PBS, fixed for 1 min in -20°C acetone (analysis grade), and the sections were rehydrated in PBS containing 0.5% BSA (BSA-PBS). Then the sections were incubated overnight at room temperature with anti-cyclin A antibody diluted 1:100 in PBS-BSA. Fluorescent photomicrographs were taken from five randomly chosen and no overlapping fields of the cyclin A-stained sections. A total of 1000 nuclei were counted per slice. The number of cyclin A positive nuclei were counted, and the positive rate was calculated. Mouse anti-rabbit cyclin A monoclonal antibody, sheep anti-mouse secondary antibody, and DAB kits were obtained from Boside Co. (Wuhan, China).

### Statistical analysis

Data are expressed as mean ± standard deviation (SD). One-way ANOVA was used for statistical comparison of the means by using SPSS statistical software. Statistical significance was set at the P < 0.05 level.

## Results

### Confirmation of chondrocytes

**Figure 1:**
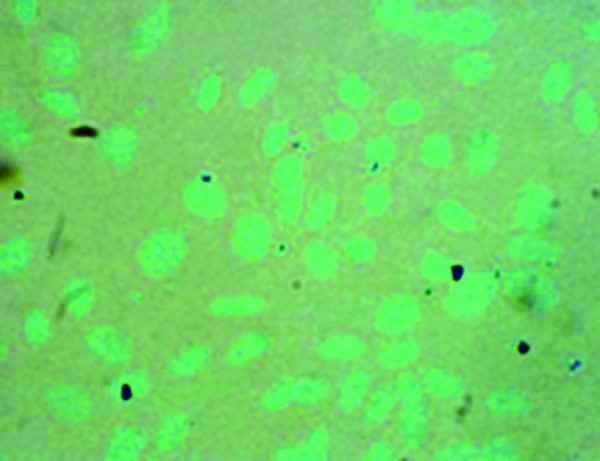
**The GAG expression in rabbit cartilage by Alcian blue staining (×600)**.

Chondrocytes were validated by GAG immunologic test. They were stained as blue by Safranin-O staining and proved to be chondrocytes (Figure 1).

### Effects of PAP and Genistein on the proliferation of chondrocytes

**Figure 2:**
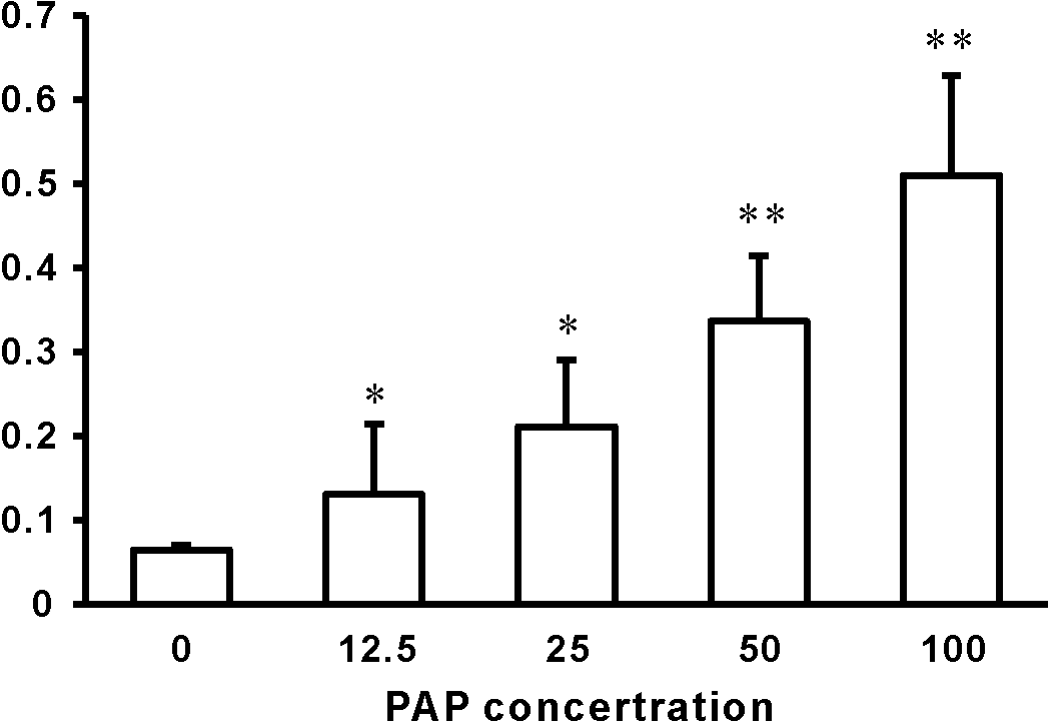
**The effect of Antler polypeptides on the proliferation of chondrocytes**. Compared to the control group, the addition of PAP significantly increased the OD values with a dose depended manner. *: P < 0.05, **: P < 0.01.

**Figure 3:**
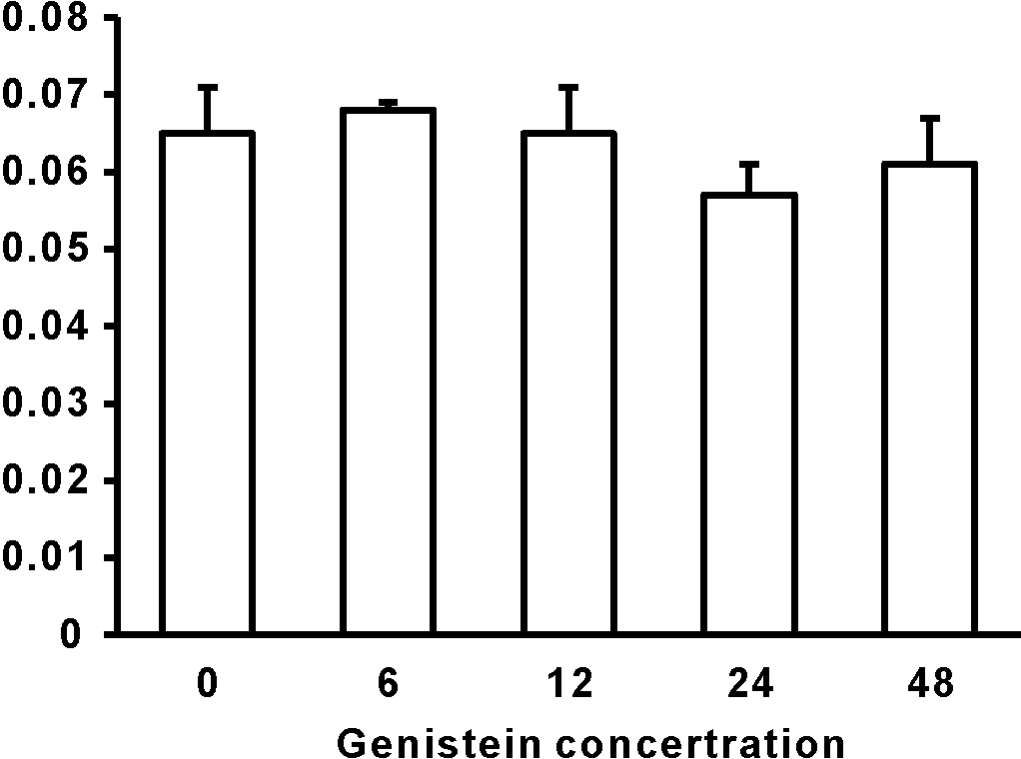
**The effect of Genistein on the proliferation of chondrocytes**. Compared to the control group, the addition of different concentrations of Genistein had no effect on the measurement of OD values.

Compared to the control group, the addition of PAP significantly increased the OD values with a dose depended manner (Figure 2). The higher concentration of PAP added, the higher OD value were observed. Since the OD value represent the number of chondrocytes, the increased OD values indicated that the addition of PAP significantly increased the number of cultured chondrocytes. However, the addition of different concentrations of Genistein had no effect on OD values (Figure 3), indicating that Genistein itself had no effect on the proliferation of chondrocytes.

### Genistein inhibited the promoting effect of PAP on chondrocytes proliferation

**Figure 4:**
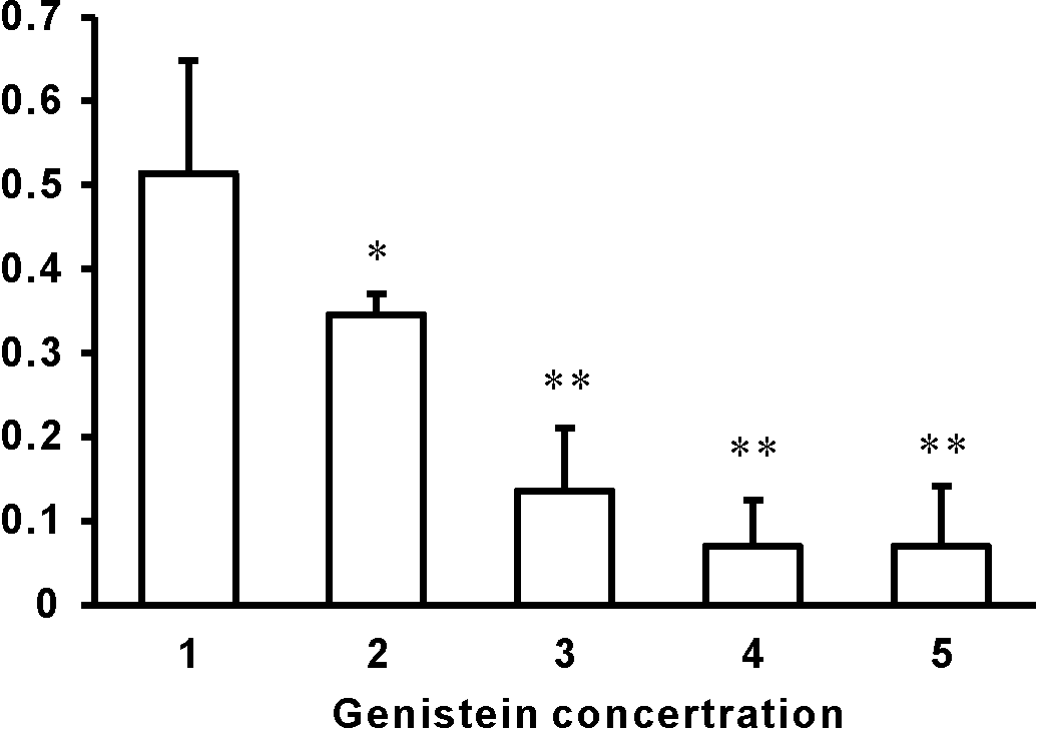
**Effect of Genistein on the promoting effect of PAP on chondrocytes proliferation**. In addition of PAP, the addition of different concentrations of Genistein significantly reduced the OD values. *: P < 0.05, **: P < 0.01.

When different concentrations of Genistein were presented, the addition of PAP (50 μg/ml) resulted in a decreased OD values (Figure 4). The more Genistein presented, the lower OD values were detected, which indicated that Genistein suppress the promoting effect of PAP on chondrocytes proliferation at a dose related manner. When the concentration of Genistein reached and beyond 24 μg/ml, the OD values detected were reduced to a very low level similar to that in control group (Figure 3), which means 24 μg/ml of Genistein could fully suppress the promoting effect of PAP on chondrocytes proliferation.

### PAP increased chondrocytes proliferation in S phase

**Figure 5:**
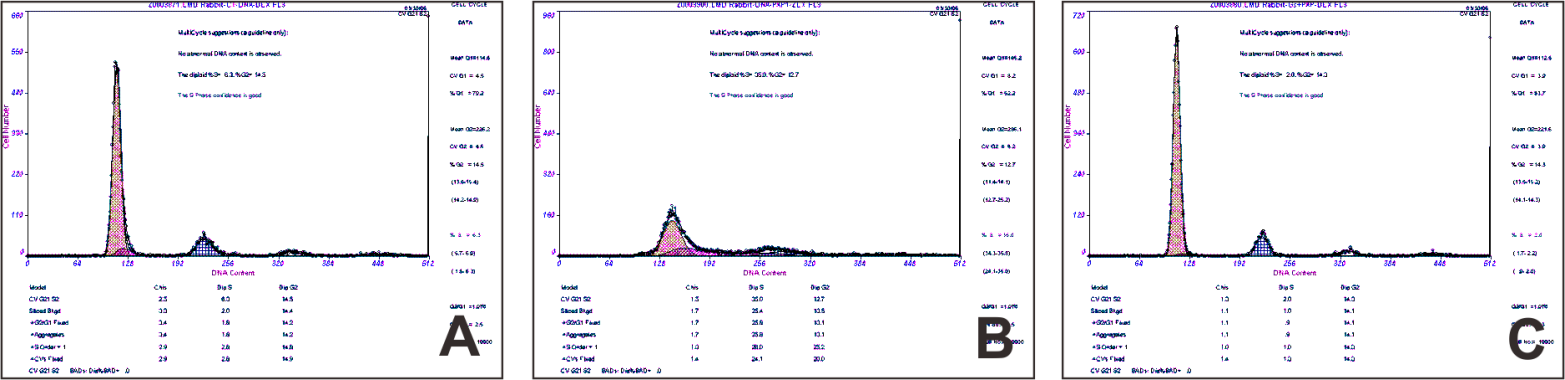
**The effect by Antler polypeptides plus Genistein on proliferation of rabbit cartilage observed by Flow cytometry**. A, the mean proportion of cells in S phase were 6.4% in control group; B, the mean proportion of cells in S phase increased to 35.2% after adding PAP; C, when Genistein was present, the proportion of cells in S phase was dramatically dropped to 4.0%.

Since the amount of DNA in cells can be measured by flow cytometry, this test was used to identify the proportions of cells in different parts of the cell cycle (the growth cycle of a cell). In this study, chondrocytes on different phases were detected using flow cytometry under different conditions, namely, in serum-free medium, with PAP, and with PAP + Genistein. Compared to control group, the mean proportion of cells in S phase increased sharply from 6.4% (Figure 5a) to 35.2% (Figure 5b) after adding PAP. However, when Genistein was present, the proportion of cells in S phase was dramatically dropped to 4.0% (Figure 5c). This result indicates that Genistein suppress the promoting effect of PAP mainly in S phase.

### PAP increased the cyclin A expression in chondrocytes

**Figure 6:**
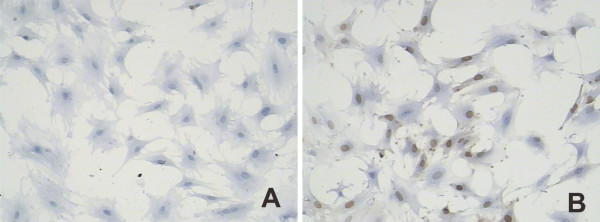
**The expression of Cyclin A by SABC immunostaining (×400)**. A, the cyclin A expression were quite few in control group; B, nearly half of the cells express cyclin A after the addition of PAP (50 μg/ml).

**Figure 7:**
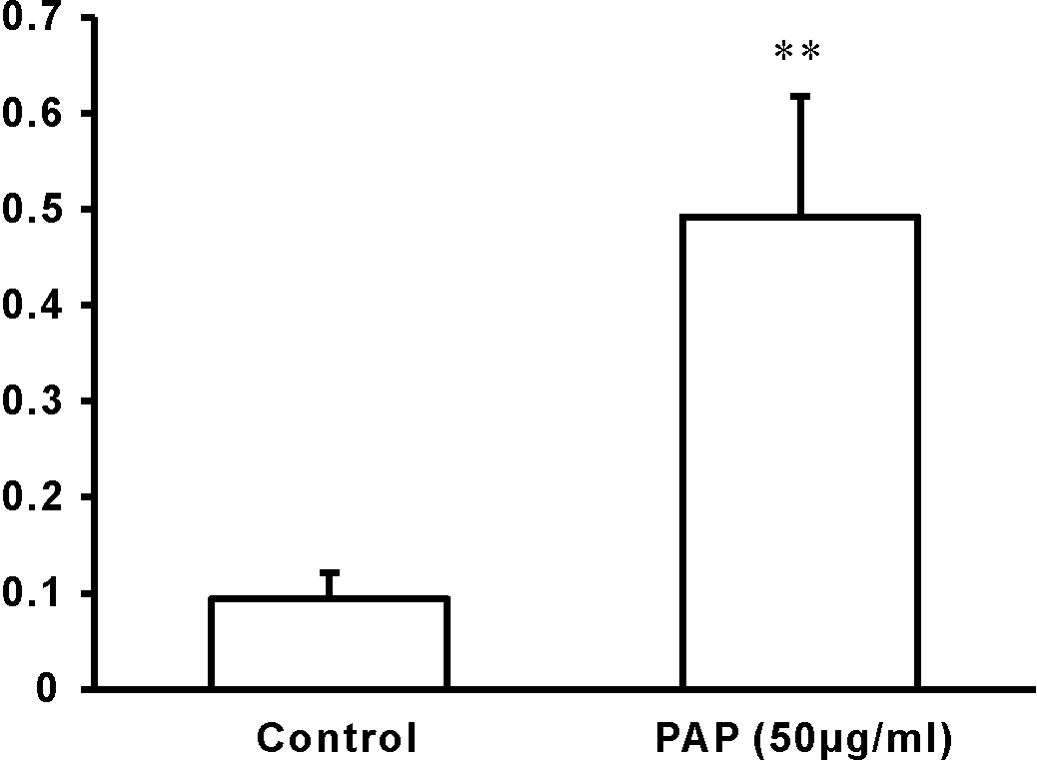
**The expression of Cyclin A by immuno-staining SABC**. Compared to the control group, the addition of PAP significantly increased the expression of Cyclin A. **: P < 0.01.

In control group, the cyclin A expression were found in one tenth of all cells, however, the cyclin A expression increased to 50% after adding PAP (50 μg/ml, Figure 6,7).

## Discussions

This report provides mechanistic insights into the promoting effects of PAP on chondrocyte proliferation and implicates the involvement of TK-signaling pathway. The proliferation of chondrocytes was evaluated directly with MTT assay, and indirectly by assessing cell cycle distribution and cyclin A expression levels when stimulated with PAP. And the involvement of TK-signaling in PAP mediating chondrocyte proliferation was identified with the treatment of genistein.

No significant difference in MTT assay, FCM or expression levels of cycling A were found between serum-free and genistein group. Indeed, the present results did not provide the evidence that genistein showed an inhibitory effect on the cultured chondrocytes with serum-free medium, suggesting that TK pathway may not be required for isolated chondrocyte proliferation.

### PAP promoted chondrocyte proliferation

With the addition of PAP, MTT assay revealed that a significant increase in number of chondrocytes compared to the serum-free cultures. Similar results have been reported by others [8]. Moreover, FCM found the significantly increased percentage proportion of chondrocytes in the S phase, indicating that PAP may accelerated S-phase entry and promote chondrocytes cell cycle progression [9]. Also, chondrocytes cultured with PAP exhibited a significant upregulation of cyclin A expression, which was consistent with the increasing percentage proportion of chondrocytes in the S phase from FCM. Since the level of cyclin A correlates directly with the proliferative state of cells [10], the data supported the hypothesis that the chondrocyte proliferation was significantly enhanced by PAP. In addition, the progressive increases in cell number, percentage of cells in S-phase and cyclin A expression levels were observed as the concentration of PAP increased from 12.5 to 100 ug/m, suggesting PAP may promote chondrocyte proliferation in a dose-dependent manner.

### Genistein inhibited the promoting effect of PAP on chondrocytes proliferation

Comparing with PAP group, there was a significant decrease in cell number in MTT assess, the percentage of cells in S-phase as well as expression levels of cyclin A in PAP plus genistein group, indicating that the promoting effects of PAP on isolated chondrocytes proliferation were reversed by the specific TK inhibitor genistein. Interestingly, genistein alone has no inhibitory effect on chondrocyte proliferation. Furthermore, progressive decreases in cell number, percentage of cells in S-phase and cyclin A expression levels were observed when the concentration of genistein increased from 6 to 24 μg/ml, demonstrating that genistein inhibited the promoting effect of PAP on chondrocyte proliferation in a dose-dependent manner. Taken together, it is reasonable to suggest that PAP promotes chondrocyte proliferation by TK-mediated signaling. Future experiment will be focused on the downstream signal pathway of TK and its regulatory mechanism(s).

## Conclusions

Our results demonstrate that chondrocyte proliferation stimulated by PAP results in an increased in the number of chondrocytes, the percentage proportion of cells in the S phase and expression of protein Cyclin A via the TK signaling pathway. Taking together, PAP promotes chondrocyte proliferation by activating TK signaling pathway.

## Competing interests

The authors declare that they have no competing interests.

## Authors' contributions

JHL carried out guarantor of integrity of the entire study; LZ carried out manuscript review; LXD carried out the molecular genetic studies, participated in the sequence alignment and drafted the manuscript; ZYW carried out statistical analysis; LC carried out immunoassays; All authors read and approved the final manuscript.
